# Precocious puberty in a rural Guatemalan boy: Unraveling the role of malnutrition and psychosocial stress

**DOI:** 10.1093/omcr/omaf150

**Published:** 2025-08-25

**Authors:** Andrea Argueta, Q Carlos Diaz, Victor Hugo Argueta, Marian Vasquez

**Affiliations:** Department of research, Universidad Francisco Marroquín, Guatemala 01011, Guatemala; Department of research, Universidad Francisco Marroquín, Guatemala 01011, Guatemala; Department of research, Universidad Francisco Marroquín, Guatemala 01011, Guatemala; Department of research, Universidad Francisco Marroquín, Guatemala 01011, Guatemala

**Keywords:** endocrinology and metabolism, radiology, oncology, psychiatry

## Abstract

Precocious puberty is a rare condition, particularly in boys, characterized by the early development of secondary sexual characteristics. This case report presents a 5-year-old male from rural Guatemala who exhibited signs of accelerated sexual maturation, including Tanner stage IV genitalia, pubic and facial hair, and advanced bone age. After evaluation, the diagnosis was associated with environmental factors such as chronic malnutrition and psychosocial stress, which likely contributed to the premature activation of the hypothalamic–pituitary-gonadal axis. In low-resource settings, factors like poverty, limited access to healthcare, and nutritional deficiencies can significantly impact growth and development, leading to atypical presentations of conditions like precocious puberty. This case highlights the importance of considering environmental factors and social determinants of health when diagnosing and managing puberty, especially in underserved populations.

## Introduction

Precocious puberty, the early onset of secondary sexual characteristics before the typical age of 8 years in girls and 9 years in boys, is a rare but significant condition that can be triggered by various factors, including endocrinological, genetic, and environmental influences. The incidence of this condition ranges from 1 in 5000 to 10 000 children [1].

**Figure 1:**
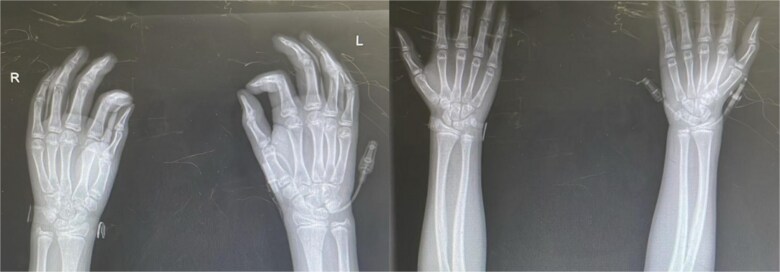
**Hands (X-ray).** Radiographic (X-ray) evidence shows advanced skeletal development, with phalangeal epiphyses appearing more developed than expected for a 5-year-old. This is consistent with the patient’s accelerated bone age, which is a key indicator of precocious puberty.

**Figure 2:**
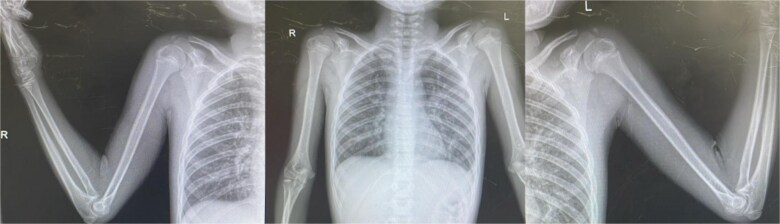
**Humerus (X-ray).** Radiographic (X-ray) evidence of the humerus reveals epiphyseal plates that are more developed than expected for the patient’s chronological age. This advanced bone maturation supports the diagnosis of precocious puberty and further correlates with the patient’s early physical development.

Precocious puberty is classified into central (gonadotropin-dependent) and peripheral (gonadotropin-independent) forms. Central precocious puberty (CPP), the most common type, results from premature activation of the hypothalamic–pituitary-gonadal (HPG) axis and is often idiopathic in girls but more likely to have a pathological cause in boys, such as hypothalamic tumors. However, growing evidence suggests that nutrition, psychosocial stress, and environmental exposures may also contribute to early pubertal onset. This case describes a 5-year-old boy from rural Guatemala with Tanner stage IV genitalia, facial and pubic hair, and rapid growth acceleration. In the absence of structural brain abnormalities or adrenal pathology, chronic malnutrition and psychosocial stress were considered potential contributors.

## Case presentation

A 5-year-old male from a rural Guatemalan community was referred to a pediatric clinic for signs of early puberty. Over six months, he experienced rapid height growth, deepening voice, and genital development beyond his age. His mother also reported increased irritability and aggression, raising concerns about behavioral changes.

The patient had a history of moderate malnutrition during his early years, common in rural areas with limited access to healthcare and nutritious food. At presentation, his weight was at the 10th percentile, and his BMI was at the 5th percentile, supporting the observation of being slightly underweight. His height, however, was at the 85th percentile for his age, indicating significant growth acceleration. Although his developmental milestones were normal until the onset of puberty, his nutritional status and environmental stressors were potential contributing factors. His mother also reported heightened family stress due to economic instability and the demands of caring for younger siblings.

On physical examination, the child was in overall good health, though underweight relative to his height. Tanner staging revealed pubic hair consistent with stage IV and testicular volumes measuring 12 ml bilaterally (measured via Prader orchidometer), confirming advanced pubertal development. Additional androgenic signs included mild facial acne and sparse axillary hair. His pubic hair was coarse and dark, distributed in a pattern typical of more advanced pubertal stages, extending toward the medial thighs. Facial hair growth was most prominent on the upper lip, with fine, pigmented hair also visible along the lateral cheeks, suggesting early terminal hair development.

As part of the diagnostic workup, a radiological assessment of bone age was performed using the Greulich and Pyle method, revealing a bone age of approximately 9 years significantly advanced compared to his chronologic age of 5 years.

To confirm the diagnosis, a series of laboratory tests were conducted. Baseline serum levels of luteinizing hormone (LH) and follicle-stimulating hormone (FSH) were elevated at 1.8 IU/l and 3.2 IU/l, respectively (drawn at 8:00 AM). Testosterone levels were also elevated at 210 ng/dl. A gonadotropin-releasing hormone (GnRH) stimulation test demonstrated a peak LH of 12 IU/l, confirming central activation of the hypothalamic–pituitary-gonadal axis. Testing for congenital adrenal hyperplasia, including 17-hydroxyprogesterone levels, was within normal limits, ruling out adrenal causes. An MRI of the brain showed no abnormalities, supporting a diagnosis of idiopathic central precocious puberty, though the role of environmental and psychosocial stressors was considered significant in this case.

Given the atypical presentation and the need to rule out genetic causes, a genetic panel was conducted, including sequencing of the MKRN3, DLK1, and KISS1R genes, which are associated with familial cases of central precocious puberty. However, given the environmental and psychosocial context, these factors were considered as potential contributors to the early pubertal onset.

Following diagnosis, the patient was referred to pediatric endocrinology and started on monthly GnRH analog therapy (leuprolide acetate 3.75 mg IM) to suppress further pubertal progression. Early follow-up at 3 and 6 weeks showed hormonal stabilization and reduction in testicular volume from 12 ml to 8 ml bilaterally. Given the psychosocial context, psychological support and nutritional counseling were also provided. The patient continues with monthly endocrinology visits, quarterly hormone monitoring, and biannual psychological assessments as part of a multidisciplinary follow-up plan.

## Discussion

Central precocious puberty (CPP) is driven by premature activation of the hypothalamic–pituitary-gonadal axis, often due to hypothalamic tumors, congenital abnormalities, or genetic mutations such as MKRN3 deficiency. Peripheral precocious puberty, in contrast, results from ectopic sex hormone production due to adrenal pathology, testicular tumors, or exogenous hormone exposure. While CPP is more commonly idiopathic in girls, boys have a higher likelihood of underlying pathology, warranting extensive evaluation. In this case, despite the absence of CNS lesions, additional factors such as chronic malnutrition, psychosocial stress, and possible endocrine disruption due to environmental exposures were considered potential contributors [2].

Several studies suggest that chronic malnutrition and psychosocial stress may contribute to early activation of the hypothalamic–pituitary-gonadal (HPG) axis, particularly in vulnerable populations. Malnutrition, while often associated with delayed puberty, can paradoxically accelerate pubertal onset in cases where early-life caloric restriction is followed by rapid catch-up growth, as observed in this patient [3]. This phenomenon may be linked to disruptions in leptin and insulin signaling, which play key roles in pubertal regulation [4]. Additionally, the psychosocial stress experienced by this child stemming from economic instability, food insecurity, and increased familial responsibilities could have triggered an early activation of the HPG axis. Stress-induced alterations in cortisol levels have been shown to modulate gonadotropin-releasing hormone (GnRH) secretion, potentially leading to premature puberty [5]. Given the absence of structural abnormalities on MRI and the lack of peripheral causes, the interplay between nutritional deficits and chronic stress emerges as a plausible contributing factor to this case of idiopathic central precocious puberty.

To ensure a comprehensive evaluation, additional testing was performed to rule out alternative causes of precocious puberty. Baseline 17-hydroxyprogesterone levels were within normal limits, effectively excluding congenital adrenal hyperplasia [6]. Thyroid function tests, including TSH and free T4, were also normal, ruling out hypothyroidism as a contributing factor. Furthermore, given the possibility of activating mutations in the LH receptor leading to gonadotropin-independent puberty, genetic testing for LHCGR mutations was considered but deemed unnecessary in the absence of suppressed gonadotropin levels. Taken together, the diagnostic approach supports a diagnosis of idiopathic central precocious puberty, with environmental and psychosocial factors likely playing a significant role in its pathogenesis [7, 8].

The role of psychosocial stress as a contributing factor to early puberty has also been increasingly recognized in recent years. There is evidence suggesting that high levels of stress, especially in resource-limited settings, can affect the timing of puberty. Stress may elevate cortisol levels, which can interfere with the normal regulation of gonadotropin-releasing hormone (GnRH), triggering early activation of the hypothalamic–pituitary-gonadal axis [4, 9]. In this case, the child’s family history of socioeconomic hardship, coupled with the stress of a large family and limited resources, likely created an environment that contributed to early puberty.

## Conclusion

This case emphasizes the importance of a comprehensive approach to diagnosing precocious puberty, considering not only common endocrinological causes but also environmental, nutritional, and psychosocial factors, particularly in resource-poor settings like rural Guatemala. By addressing these contributing factors, clinicians can improve diagnosis and management, ensuring that children in underserved regions receive the appropriate care to navigate the challenges associated with early puberty.

## Consent

Written informed consent was obtained prior to the inception of this case. As the patient is a minor, the consent form was signed by his legal guardian.

## Guarantor

Victor Hugo Argueta.

## Data Availability

All data supporting this case report are included in the article, with no additional data available due to patient confidentiality.
